# Non-invasive ventilation for preoxygenation during prehospital anaesthesia – a prospective observational study

**DOI:** 10.1186/s13049-025-01386-3

**Published:** 2025-04-23

**Authors:** Harry Ljungqvist, Jouni Nurmi

**Affiliations:** Emergency Medicine and Services, University of Helsinki and Helsinki University Hospital, Helsinki, Finland

**Keywords:** Air ambulances, Emergency medical services, Critical care, Rapid Sequence Induction and Intubation, Anaesthesiology, Noninvasive ventilation

## Abstract

**Background:**

Preoxygenation is used to prevent hypoxia during anaesthesia and intubation. In a prehospital setting, preoxygenation is usually performed using a non-rebreather mask or a bag-valve-mask. These methods are however not sufficient for some critically ill patients. Non-invasive ventilation (NIV) has been shown to be more effective than other methods for preoxygenation of these patients in a hospital setting. Despite this, the use of NIV for preoxygenation has not been reported in a prehospital setting. The purpose of this study is to describe the prehospital use of, and experience with, NIV as a preoxygenation technique in patients undergoing prehospital emergency anaesthesia (PHEA).

**Methods:**

In this prospective observational study, we included 42 patients preoxygenated with NIV for PHEA by one Finnish helicopter emergency medical services unit. We gathered data on, among other things, patient characteristics, vital signs, success of preoxygenation, post-intubation complications and mortality. In addition, we conducted a semi-structured survey on experiences of the use of NIV for preoxygenation among the prehospital physicians in the study unit. Descriptive analyses were performed as well as calculating confidence intervals.

**Results:**

During the study period from October 2022 to May 2023, a total of 115 PHEAs were performed and NIV preoxygenation was used in 42 (*n* = 42/115, 37%) of these. Preoxygenation using NIV was technically successful in 100% of cases (*n* = 42/42, 95% CI 92—100). The median (IQR) oxygen saturation at HEMS arrival was 98% (95—99) and preoxygenation with NIV achieved a median (IQR) oxygen saturation post-intubation of 99% (97—100). No complications of hypoxia were documented, and the rate of pneumonia and mortality did not exceed what was expected based on literature. In the survey, 40% (*n* = 4/10) of physicians reported using NIV routinely for all patients while 60% (*n* = 6/10) only used it for those considered susceptible to desaturation.

**Conclusions:**

This study demonstrates that NIV for preoxygenation has been implemented and is frequently used in prehospital settings in Finland, and that the intervention seems technically successful without clear adverse events.

**Supplementary Information:**

The online version contains supplementary material available at 10.1186/s13049-025-01386-3.

## Background

Prehospital emergency anaesthesia (PHEA) is a standard treatment for critically ill and major trauma patients [1, 2]. Anaesthesia-induced apnoea disposes the patient to one of the most common adverse events during the intubation of critically ill patients: desaturation and hypoxia, which may further lead to complications such as cardiovascular collapse, anoxic brain injury and death [3]. To delay the time to desaturation and prevent possible additional complications, it is crucial to perform adequate preoxygenation [4]. This is achieved in theory by the denitrogenation of alveoli so that the functional residual capacity of the lungs serves as an oxygen reserve during apnoea [4]. In a prehospital setting, preoxygenation is usually performed using a non-rebreather mask or bag-valve-mask (BVM), possibly with adjuncts such as nasal cannulas [5].

However, these techniques might not be sufficient for critically ill patients with an elevated alveolar-arterial gradient or ventilation-perfusion mismatch, for example a patient with acute respiratory distress syndrome. In these patients, even a well-executed denitrogenation does not provide a reservoir of oxygen large enough to prevent desaturation [4]. Non-invasive ventilation (NIV) has been shown to improve the preoxygenation of these critically ill patients in hospital settings [6–8]. While prehospital critical care units have been equipped with portable ventilators for controlled ventilation after intubation, their use in preoxygenation has not been reported [9].

The purpose of this study is to describe the indications, success and outcomes of using NIV as a prehospital preoxygenation technique. Prospective clinical data on the use of NIV for preoxygenation were collected and complemented by a survey of the prehospital physicians.

## Methods

### Study design

This study was an investigator-initiated, prospective, single centre, observational descriptive study of the use of NIV as a preoxygenation technique. The study was approved and access to patient records was granted by Helsinki University Hospital (HUS/280/2019 §9). Informed consent and ethical approval were not deemed necessary as it was an observational study not affecting patient treatment. The study consisted of chart reviews that were further complemented by a survey among the physicians working in the HEMS unit during the same period.

### Setting

This study was conducted in one Finnish helicopter emergency medical services (HEMS) unit in the southern part of Finland. The unit provides prehospital critical care, including PHEA, to a population of approximately 1.3 million in an area of 10,000 square km [10]. The HEMS unit is staffed with an anaesthesiologist-led team including a pilot and a crew member. The unit is dispatched by the emergency dispatch centre as an addition to ground-based units in both medical and traumatic emergencies. The role of the HEMS unit is to provide advanced critical care to patients as well as facilitate faster transport by helicopter for time-critical patients. The unit is dispatched around 2,500 times, and performs PHEA about 250 times, yearly with a highly standardized process [11]. The medical responsibility of the treatment and medical staff is carried by the local hospital (Helsinki University Hospital, HUS). More information about the Finnish HEMS system can be found in a recently published article [10].

### Participants, data collection and variables

The first part of the study included patients of all ages and aetiologies who received preoxygenation with NIV prior to PHEA by HEMS. Data was gathered between 15 th October 2022 and 5 th May 2023. The physicians in charge of patient care were advised to collect prehospital patient records into a separate folder after NIV had been used for preoxygenation. When recruitment was over, the cases were then collected by the first author and a chart review of both prehospital and in-hospital patients records was done. Data gathered included patient characteristics, vital parameters, the airway management process, the oxygen saturation throughout the prehospital phase, radiology reports and information on patient outcomes.

Vital parameters, including oxygen saturation of the patients, were monitored using either Lifepak 15 (Physio-Control, WA, USA), CorPuls3 (Corpuls, Kaufering, Germany) or Zoll X series (Zoll Medical Corporation, MA, USA). Data from these devices are automatically transferred to the electronic patient record system (Merlot Medi, CGI, Helsinki, Finland). Data on possible pneumonia was gathered from the radiology reports (first 24 h as well as within 5 days) and was defined as present if the radiologist mentioned pneumonia or a suspicion of pneumonia. We included chest x-rays but also potential other imaging modalities like computer tomography of the chest. Mortality was defined as death before discharge from Helsinki University Hospital. The body mass index (BMI) was gathered from the electronic patient records when available – we used the first weight and length measurements documented during the hospital stay but accepted any records as long as they were taken during the same admission. The international established quality indicators for prehospital advanced airway management provided definitions of hypoxia (new < 90% oxygen saturation during airway management) and first pass success (successful tracheal intubation on first passing of the laryngoscope blade past the front teeth) [12]. Technical success of the use of NIV for preoxygenation was defined as no need to change method during the process of anaesthesia.

The second part of the study was included to further evaluate the intervention and involved a survey consisting of 16 questions (supplementary material A). The survey was distributed on 29 th September 2023 among the physicians working in the studied unit and the last survey was returned on 17 th October 2023. The survey followed a structured form consisting of single choice and multiple-choice questions as well as some short open-ended questions and was designed based on expert opinion and preliminary analysis of the quantitative data.

### Prehospital anaesthesia and preoxygenation

The study unit performs PHEA according to a comprehensive standard operating procedure dictating, among other things, the routine use of a video laryngoscope, bougie and checklist as well as standardising the communication. More details of the techniques, implementation and success of the standard operating procedure have been presented in recent publications [11, 13].

The method of preoxygenation has, however, been left to the discretion of the physician. The standard method is a BVM with a reservoir bag and 15 L/min oxygen flow, either by spontaneous breathing or manually supported positive pressure ventilation. At the time of the study, nasal cannula was allowed as an adjunct. The HEMS team has been carrying portable ventilators primarily used for controlled ventilation after intubation. The use of the ventilator to provide NIV during preoxygenation has associated training and is defined by the standard operating procedure, but the indications for using this method are not strict.

**Fig. 1:**
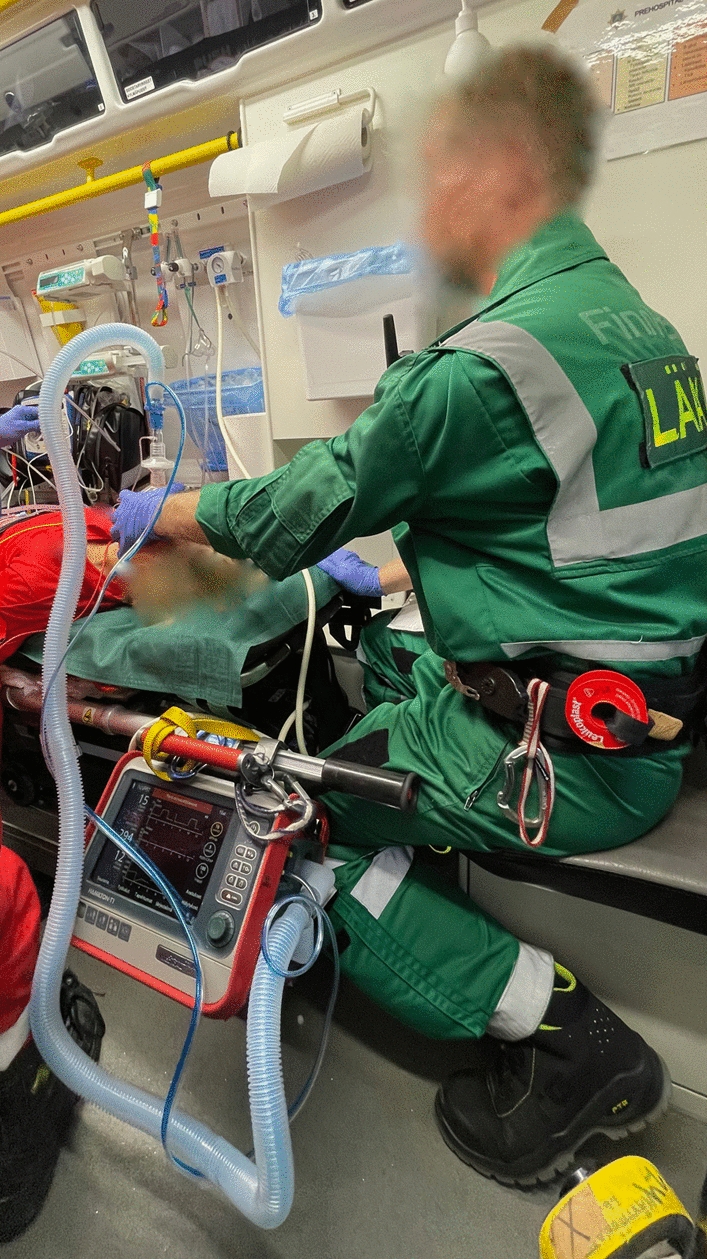
Non-invasive ventilation in use as preoxygenation method

In the standardised NIV preoxygenation, a Hamilton- T1 (Hamilton-Medical, Graubünden, Switzerland) portable ventilator is connected to a standard ventilation mask with normal hosing (the same as for the BVM) instead of a specific NIV mask. The mask is held with a two-hand grip to provide a tight seal (Fig. 1). The spontaneous/timed non-invasive ventilation (NIV-ST) mode is used with 100% inspired oxygen, 5 cmH2O positive end expiratory pressure (PEEP) and 15 cmH2O (10 cmH2O above PEEP) inspiratory pressure support ventilation (PSV). The triggering sensitivity is set to 3 L/min and ventilation frequency to 12 breaths per minute. In this mode, the ventilator supports the spontaneous breaths and further through mandatory positive pressure ventilation ensures a ventilation frequency of at least 12.

### Statistical methods

As this was an observational descriptive study, mostly simple descriptive analyses were performed. Continuous variables are presented as median and interquartile range (IQR) or range, while categorical variables are presented as numbers and percentages (%). Confidence intervals (CI) were calculated using the prop.test function. All analyses were performed using the R (R Core Team, 2023. R Foundation for Statistical Computing, Vienna, Austria) language and RStudio (Posit team, 2023. Posit Software, PBC, Boston, MA, USA) with the following packages: stats, tidyverse and gtsummary [14, 15].

## Results

Incidence, patient characteristics and airway management.

During the study period, 15 th October 2022 to 5 th May 2023, the HEMS unit encountered 424 patients and 115 of them underwent PHEA. NIV was used for preoxygenation in 42 cases (*n* = 42/115, 37%). Most of the patients had some acute intracranial pathology (Table 1).

**Table 1:** Patient characteristics

Characteristic	*n* = 42	Missing data, n
Patient age	59 (45—74)	1
*Sex*		0
Female	19 (45%)	
Male	23 (55%)	
Body Mass Index	25.1 (23.1—28.2)	6
*Patient category*		0
Infection	1 (2.4%)	
Intoxication	8 (19%)	
Unconscious	1 (2.4%)	
Cardiac arrest	4 (9.5%)	
Seizure	8 (19%)	
Stroke	11 (26%)	
Traumatic brain injury	9 (21%)	
*Vital signs at EMS arrival*		
Heart rate	89 (69—117)	9
Systolic blood pressure	140 (122—160)	11
Oxygen saturation	91 (82—97)	8
Glasgow Coma Scale	3.00 (3.00—6.00)	9
Respiratory rate	18 (12—26)	24

Numbers are presented as n (%) and median (IQR). EMS, emergency medical services

None of the physicians reported any specific indication for using NIV for preoxygenation. Propofol was used for induction in 23 patients (*n* = 23/42, 55%) with a median (IQR) dose of 70 mg (50–100), and ketamine was used in the remaining 19 patients (*n* = 19/42, 45%) with a median (IQR) dose of 50 mg (50–63). Rocuronium was used as a neuromuscular blocking agent in all 42 patients (*n* = 42/42, 100%) with a median (IQR) dose of 100 mg (80–100), and fentanyl was used as an adjunct in 29 patients (*n* = 29/42, 69%) with a median (IQR) dose of 0.25 mg (0.20–0.25). First pass success was achieved in all 42 patients (*n* = 42/42, 100%).

### Preoxygenation

**Fig. 2:**
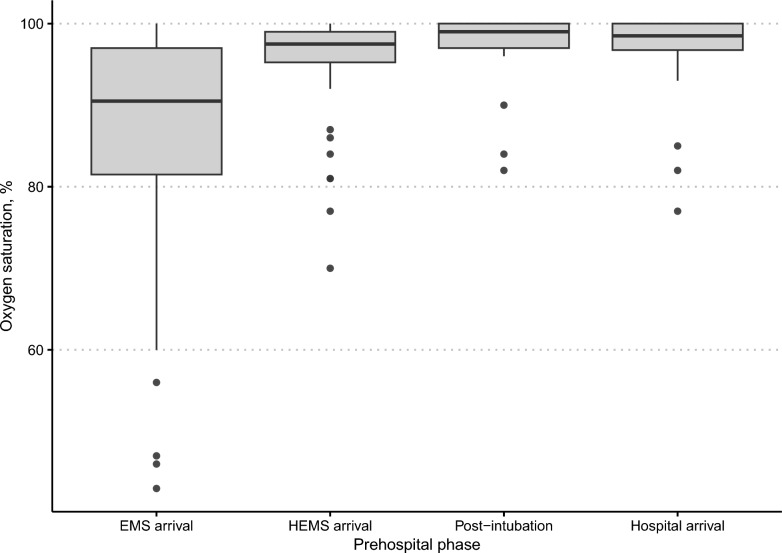
Oxygen saturation during the prehospital phase

All preoxygenations using NIV were technically successful as in no need for a change of preoxygenation method (*n* = 42/42, 100%, 95% CI 92–100). Fourteen patients (*n* = 14/34, 41%, missing data = 8) were hypoxic (oxygen saturation < 90%) when the first unit arrived but interventions prior to HEMS arrival corrected all but six cases (*n* = 6/34, 18%, missing data = 8). The median (IQR) oxygen saturation at HEMS arrival was 98% (95–99). Two cases were hypoxic after intubation; however, they were hypoxic throughout the prehospital phase and NIV preoxygenation was not able to correct this. The first one suffered from a severe pneumonia which could explain the difficulties, the second one had no clear findings that could allude to the aetiology of the hypoxia. Thus, no complications of hypoxia as dictated by the established quality indicators occurred [12]. Preoxygenation with the use of NIV achieved a median (IQR) oxygen saturation post-intubation of 99% (97–100) which, for all but three patients, remained above 94% during the rest of the prehospital phase (median oxygen saturation at hospital arrival 98.5%, 97–100) (Fig. 2).

The box plot presents median (thick line) and interquartile range (box) and 1.5 * IQR (whiskers), while the dots represent individual outliers. EMS, emergency medical services; HEMS, helicopter emergency medical services.

### In-hospital treatment and outcomes

All patients survived to hospital handover (*n* = 42/42, 100%) with 92% (*n* = 35/38, 92%, missing data = 4) admitted to the ICU. Thirty-five patients had a chest x-ray done within 24 h of arrival and six of these cases (*n* = 6/35, 17%) had a suspected aspiration pneumonia noted on the radiology report. No cases of pneumothorax or other traumatic pathologies possibly related to preoxygenation was seen in the x-rays. The in-hospital mortality of these patients was 29% (*n* = 12/41, missing data = 1), with 29 surviving to discharge from Helsinki University Hospital (Table 2).

**Table 2:** – Patient outcomes

Outcomes	*n* = 42	Missing data, n
Pneumonia on initial x-ray	6 (17%)	7
Pneumonia within five days	12 (31%)	3
Length of ventilator stay	1 (1—3)	4
Length of intensive care	2.5 (1.0—4.8)	4
Length of hospital stay	8 (4—25)	17
In-hospital mortality	12 (29%)	1

Numbers are presented as n (%) and median (IQR). The unit of length is ‘days’. The missing values for the length of hospital stay includes both missing values and mortality before discharge

### Results of the survey

During the study period, there were ten physicians working in the HEMS unit and all ten answered the questionnaire. They had a median of 14 years (range 6 −27) experience in anaesthesiology and a median of 9.5 years (range 2–21) of experience in prehospital critical care.

All physicians reported having used NIV for preoxygenation with experience ranging from 1 to 50 cases. The thoughts on indication were divided with four (*n* = 4/10, 40%) using it routinely for all patients while the rest (*n* = 6/10, 60%) left it for cases of severe hypoxia or patients assumed to rapidly desaturate (free text entries included: *“obese”, “severe pneumonia”, “suspected atelectasis”*). However, two (*n* = 2/6, 33%) who reported not using NIV routinely additionally mentioned that it probably should be used for all patients. Regarding contra-indications for NIV preoxygenation, the consensus was that NIV was not suitable for patients with excessive vomiting, bleeding in upper airway, high risk of aspiration as well as facial trauma.

In evaluating the sufficiency of preoxygenation, seven (*n* = 7/10, 70%) physicians reported using a minimum time of three minutes and two of these further reported observing the peripheral oxygen saturation as well. The remaining three (*n* = 3/10, 30%) reported relying on the preparations for intubation taking enough time to achieve sufficient preoxygenation.

## Discussion

### Main findings

This is, to our knowledge, the first prospective study of the use of NIV in prehospital preoxygenation. No strict indications for preoxygenation with NIV were defined in the standard operating procedure for PHEA and the method was used by the HEMS physicians on a varied population consisting of mostly neurological patients without sever respiratory compromises. All cases were technically successful and, while optimal oxygen saturation from NIV preoxygenation was not achieved in two cases, no complications of desaturation during intubation were observed. The incidence of suspected aspiration pneumonia and the rate of mortality did not raise concerns regarding the preoxygenation method.

### Indications for NIV preoxygenation

A key factor for the safety of the apnoeic phase of anaesthesia and intubation is how successful preoxygenation is [16]. The conventional method when preoxygenating a critically ill prehospital patient is to denitrogenate the lung volume using the patient’s own spontaneous respirations while providing a high fraction of inspired oxygen [9]. However, in patients with an elevated alveolar-arterial gradient, a ventilation-perfusion mismatch and decreased functional residual capacity, this might not be sufficient [4, 16]. For these pathologies, NIV might provide a solution as it utilises PSV in combination with PEEP. This combination has been shown to result in more effective alveolar recruitment and subsequent decreased shunt fraction [6, 8]. The use of assisted inspiratory support and PEEP is also identified as the best method for prehospital preoxygenation in the recently published quality indicators for PHEA [12].

Previous studies on the use of NIV for preoxygenation have mainly focused on patients with severe hypoxia or obesity [6, 8, 17, 18]. These patients are especially susceptible to desaturation and thought to benefit the most from preoxygenation using NIV. The population in our study, however, consisted mainly of patients with neurological pathologies and the majority did not suffer from obesity. Further, almost all already had a relatively high oxygen saturation prior to preoxygenation. Thus, our study population potentially benefitted less from NIV preoxygenation and might not be comparable to other studies of the topic. However, in healthy volunteers, NIV also achieved a better fraction of expired oxygen in comparison to both BVM and the non-rebreather mask [5]. No specific reason for the use of NIV for preoxygenation was reported in our cases but in the survey, 60% of the physicians reported that they would use NIV in patients that are predicted to easily desaturate during intubation, like obese and hypoxic patients, while the rest used it routinely for all patients. Whether NIV is safe and could or should be used routinely for preoxygenation in all patients warrants further study.

### Performing preoxygenation using non-invasive ventilation

The physiological benefits of using NIV for preoxygenation originate partly from the rise in airway pressure that increases functional residual capacity and opens atelectasis. The NIV-ST mode of the ventilator used in the study increases airway pressure both during inspiration and expiration. The settings used in this study largely correspond to those described previously, although higher peak pressures have also been used [6, 19–21]. The choice of settings is not inconsequential, as the pressure applied to the airway by the ventilator might expose the patient to gastric insufflation, potentially followed by regurgitation and aspiration [22]. However, some studies have found this concern to be unfounded, even favouring NIV over other methods regarding risk of aspiration [7, 19]. The use of NIV in combination with a tight mask seal also potentially enables a higher fraction of inspired oxygen to be provided to the patient in comparison to non-rebreather masks and most BVM systems [23, 24]. Another area of concern has been ventilation during induction and in our study the ventilator was set to perform mandatory ventilations at a rate of 12 breaths per minute in case of loss of spontaneous breathing. A recent study of ventilations during induction of critically ill patients using a BVM presented a higher oxygen saturation and less hypoxia with ventilations without any significant increase in aspiration or pneumonia [25].

There are further potential benefits, beyond physiological, of using a ventilator for preoxygenation. The ventilator can be well synchronized with the spontaneous breathing of the patient and seamlessly move to mandatory ventilation as spontaneous ventilation ceases after the induction of anaesthesia. This will shorten the apnoeic phase as well as lower the risk of a premature removal of the tight mask before apnoea and subsequent loss of preoxygenation [26]. The ventilator will also allow the provider to maintain a two-handed grip on the mask, allowing for a tighter seal and improved manual airway manipulation compared to using a BVM where one hand needs to perform potential manual ventilations [21]. The ventilator also provides a lower peak airway pressure during ventilation as well as prevents hyperventilation, both shown to be a risk when using a manual BVM [27, 28].

### Success of preoxygenation and outcomes of the patients

Evaluating the sufficiency of the preoxygenation in a clinical situation is difficult. Guidelines suggest using end-tidal oxygen partial pressure to assess the preoxygenation [29]. However, the equipment to measure this is not widely available in prehospital settings. In the survey, the physicians reported using time, striving for a minimum duration of 3 min, and the peripheral oxygen saturation to evaluate preoxygenation. Both oxygen saturation and time are far from perfect measurements of adequate preoxygenation. The oxygen saturation of the peripheral haemoglobin is unable convey the level of denitrogenation performed, and while a lengthy preoxygenation might result in adequate denitrogenation, this may not be true in a critically ill hypoxic patient [4]. Furthermore, the optimal duration of NIV to recruit additional lung volume in the context of preoxygenation is unknown.

The rate of post-intubation hypoxia during PHEA in the whole Finnish HEMS system has been reported to be 4.3%, which is largely the same as in our sample [30]. Severe hypoxia, defined as oxygen saturations below 80%, has been reported to occur at a rate of 9–26% during emergency anaesthesia inside the hospital [31]. No such cases were seen in this study. The rate of complications, primarily hypoxia due to prolonged intubation, increases for every subsequent intubation attempt and thus, first pass success is a key confounder in studies of preoxygenation [32]. All patients in this study were intubated on the first attempt, which is likely to decrease the risk of complications. This finding works both ways as optimal preoxygenation also might increase the time to perform an intubation attempt, thus could improve first pass success as desaturation is a common reason to abort an attempt [33]. Furthermore, NIV has been shown to have potential longer term positive effects on oxygen saturation levels, something also seen in our patients where oxygen saturation remained relatively stable throughout the prehospital phase [6].

The PEEP and PSV as well as the positive pressure ventilation applied by the ventilator did not seem to cause exceptionally high levels of pneumonia, with the 5% seen in our patients. It is lower than the 28.3% reported in a large in-hospital study of NIV for preoxygenation but our definitions and study designs are not uniform [8]. Ventilator-associated pneumonia occurs within five days in 5–40% of patients on mechanical ventilation; comparable levels were seen in our small sample as well [34]. The in-hospital and 30-day mortality were quite high in our study but due to a small sample size and lack of control group, this should not be interpreted as an effect of the preoxygenation method but more a reflection of the overall morbidity of the patients.

### Strengths and limitations

This was an observational study in conjunction with a small survey and thus, extrapolation should be performed with care. Further, this study does not include any control group nor is the sample size sufficient to present robust data on the safety of the intervention. However, the evidence of the benefit of NIV in a hospital environment is established, and no severe safety concerns have been raised in previous publications. Thirdly, the data in this study is partly based on non-structured text entries and thus is susceptible to bias. Fourthly, our patient cohort consisted mostly of patients with neurological ailments and with a low rate of severe respiratory compromises, which may not be a patient group that most benefit from NIV preoxygenation. A final strength is that in-hospital personnel were unaware of the study and thus, the radiology reports could be seen as blinded, for example.

## Conclusion

The results of this study demonstrate that NIV for preoxygenation has been implemented in a prehospital critical care setting, and the intervention seems technically successful without any clear signals of adverse events. Further research is needed on the efficacy and safety of the intervention in this setting and whether it is indicated for all patients or should be left to those deemed most in need.

## Supplementary Information

Additional file 1.

## Data Availability

The datasets used and analysed during this study are available from the corresponding author on a reasonable request.

## Abbreviations

BMI: Body mass index
BVM: Bag-valve-mask
CI: Confidence intervals
EMS: Emergency medical services
HEMS: Helicopter emergency medical services
HUS: Helsinki University Hospital
IQR: Interquartile range
NIV: Non-invasive ventilation
NIV-ST: Spontaneous / timed non-invasive ventilation
PEEP: Post expiratory end pressure
PHEA: Prehospital emergency anaesthesia
PSV: Pressure support ventilation

## References

[1] Lockey DJ, Crewdson K, Davies G, Jenkins B, Klein J, Laird C, et al. AAGBI: Safer pre-hospital anaesthesia 2017. Anaesthesia. 2017;72:379–90. 28045209 10.1111/anae.13779 PMC5324693

[2] Rehn M, Hyldmo PK, Magnusson V, Kurola J, Kongstad P, Rognås L, et al. Scandinavian SSAI clinical practice guideline on pre-hospital airway management. Acta Anaesthesiol Scand. 2016;60:852–64. 27255435 10.1111/aas.12746 PMC5089575

[3] Mosier JM, Joshi R, Hypes C, Pacheco G, Valenzuela T, Sakles JC. The Physiologically Difficult Airway. West J Emerg Med. 2015;16:1109–17. 26759664 10.5811/westjem.2015.8.27467 PMC4703154

[4] Mosier JM, Hypes CD, Sakles JC. Understanding preoxygenation and apneic oxygenation during intubation in the critically ill. Intensive Care Med. 2017;43:226–8. 27342820 10.1007/s00134-016-4426-0

[5] Groombridge CJ, Ley E, Miller M, Konig T. A prospective, randomised trial of pre-oxygenation strategies available in the pre-hospital environment. Anaesthesia. 2017;72:580–4. 28295147 10.1111/anae.13852

[6] Baillard C, Fosse J-P, Sebbane M, Chanques G, Vincent F, Courouble P, et al. Noninvasive Ventilation Improves Preoxygenation before Intubation of Hypoxic Patients. Am J Respir Crit Care Med. 2006;174:171–7. 16627862 10.1164/rccm.200509-1507OC

[7] Fong KM, Au SY, Ng GWY. Preoxygenation before intubation in adult patients with acute hypoxemic respiratory failure: a network meta-analysis of randomized trials. Crit Care. 2019;23:319. 31533792 10.1186/s13054-019-2596-1 PMC6751657

[8] Gibbs KW, Semler MW, Driver BE, Seitz KP, Stempek SB, Taylor C, et al. Noninvasive Ventilation for Preoxygenation during Emergency Intubation. N Engl J Med. 2024;390:2165–77. 38869091 10.1056/NEJMoa2313680 PMC11282951

[9] Boulton AJ, Mashru A, Lyon R. Oxygenation strategies prior to and during prehospital emergency anaesthesia in UK HEMS practice (PREOXY survey). Scand J Trauma Resusc Emerg Med. 2020;28:99. 33046111 10.1186/s13049-020-00794-x PMC7552361

[10] Saviluoto A, Björkman J, Olkinuora A, Virkkunen I, Kirves H, Setälä P, et al. The first seven years of nationally organized helicopter emergency medical services in Finland – the data from quality registry. Scand J Trauma Resusc Emerg Med. 2020;28:46. 32471467 10.1186/s13049-020-00739-4 PMC7260827

[11] Ångerman S, Kirves H, Nurmi J. Multifaceted implementation and sustainability of a protocol for prehospital anaesthesia: a retrospective analysis of 2115 patients from helicopter emergency medical services. Scand J Trauma Resusc Emerg Med. 2023;31:21. 37122004 10.1186/s13049-023-01086-w PMC10148755

[12] Kottmann A, Krüger AJ, Sunde GA, Røislien J, Heltne J-K, Carron P-N, et al. Establishing quality indicators for prehospital advanced airway management: a modified nominal group technique consensus process. Brit J Anaesth. 2021; 10.1016/j.bja.2021.08.031 PMC8792832 34674835

[13] Ångerman S, Kirves H, Nurmi J. A before-and-after observational study of a protocol for use of the C-MAC videolaryngoscope with a Frova introducer in pre-hospital rapid sequence intubation. Anaesthesia. 2018;73:348–55. 29315473 10.1111/anae.14182

[14] Sjoberg DD, Whiting K, Curry M, Lavery JA, Larmarange J. Reproducible Summary Tables with the gtsummary Package. R J. 2021;13:570.

[15] Wickham H, Averick M, Bryan J, Chang W, McGowan L, François R, et al. Welcome to the Tidyverse. J Open Source Softw. 2019;4:1686.

[16] Natt BS, Malo J, Hypes CD, Sakles JC, Mosier JM. Strategies to improve first attempt success at intubation in critically ill patients. Br J Anaesth. 2016;117:i60–8. 27221259 10.1093/bja/aew061

[17] Delay J-M, Sebbane M, Jung B, Nocca D, Verzilli D, Pouzeratte Y, et al. The Effectiveness of Noninvasive Positive Pressure Ventilation to Enhance Preoxygenation in Morbidly Obese Patients&colon. A Randomized Controlled Study Anesth Analg. 2008;107:1707–13. 18931236 10.1213/ane.0b013e318183909b

[18] Futier E, Constantin J-M, Pelosi P, Chanques G, Massone A, Petit A, et al. Noninvasive Ventilation and Alveolar Recruitment Maneuver Improve Respiratory Function during and after Intubation of Morbidly Obese Patients. Anesthesiology. 2011;114:1354–63. 21478734 10.1097/ALN.0b013e31821811ba

[19] Baillard C, Prat G, Jung B, Futier E, Lefrant JY, Vincent F, et al. Effect of preoxygenation using non-invasive ventilation before intubation on subsequent organ failures in hypoxaemic patients: a randomised clinical trial. Br J Anaesth. 2018;120:361–7. 29406184 10.1016/j.bja.2017.11.067

[20] Frat J-P, Ricard J-D, Quenot J-P, Pichon N, Demoule A, Forel J-M, et al. Non-invasive ventilation versus high-flow nasal cannula oxygen therapy with apnoeic oxygenation for preoxygenation before intubation of patients with acute hypoxaemic respiratory failure: a randomised, multicentre, open-label trial. Lancet Respir Med. 2019;7:303–12. 30898520 10.1016/S2213-2600(19)30048-7

[21] Grant S, Khan F, Keijzers G, Shirran M, Marneros L. Ventilator-assisted preoxygenation: Protocol for combining non-invasive ventilation and apnoeic oxygenation using a portable ventilator. Emerg Med Australas. 2016;28:67–72. 26764895 10.1111/1742-6723.12524

[22] He G, Ma L, Tian K, Cao Y, Qin Z. Effect of facemask oxygenation with and without positive pressure ventilation on gastric volume during anesthesia induction in patients undergoing laparoscopic cholecystectomy or partial hepatectomy: a randomized controlled trial. BMC Anesthesiol. 2022;22:412. 36581835 10.1186/s12871-022-01958-1 PMC9801608

[23] Quintana S, Pérez JM, Alvarez M, Vila JS, Jara F, Nava JM. Maximum FIO2 in minimum time depending on the kind of resuscitation bag and oxygen flow. Intens Care Med. 2004;30:155–8. 10.1007/s00134-003-2010-x 14551682

[24] Weingart SD, Levitan RM. Preoxygenation and Prevention of Desaturation During Emergency Airway Management. Ann Emerg Med. 2012;59:165-175.e1. 22050948 10.1016/j.annemergmed.2011.10.002

[25] Casey JD, Janz DR, Russell DW, Vonderhaar DJ, Joffe AM, Dischert KM, et al. Bag-Mask Ventilation during Tracheal Intubation of Critically Ill Adults. New Engl J Med. 2019;380:811–21. 30779528 10.1056/NEJMoa1812405 PMC6423976

[26] Mosier J, Reardon RF, DeVries PA, Stang JL, Nelsen A, Prekker ME, et al. Time to Loss of Preoxygenation in Emergency Department Patients. J Emerg Med. 2020;59:637–42. 32771321 10.1016/j.jemermed.2020.06.064

[27] von Goedecke A, Voelckel WG, Wenzel V, Hörmann C, Wagner-Berger HG, Dörges V, et al. Mechanical Versus Manual Ventilation via a Face Mask During the Induction of Anesthesia&colon; A Prospective, Randomized. Crossover Study Anesth Analg. 2004;98:260–3. 14693633 10.1213/01.ANE.0000096190.36875.67

[28] Herriger A, Frascarolo Ph, Spahn DR, Magnusson L. The effect of positive airway pressure during pre-oxygenation and induction of anaesthesia upon duration of non-hypoxic apnoea. Anaesthesia. 2004;59:243–7. 14984521 10.1111/j.1365-2044.2004.03615.x

[29] Higgs A, McGrath BA, Goddard C, Rangasami J, Suntharalingam G, Gale R, et al. Guidelines for the management of tracheal intubation in critically ill adults. Brit J Anaesth. 2018;120:323–52. 29406182 10.1016/j.bja.2017.10.021

[30] Ljungqvist H, Pirneskoski J, Saviluoto A, Setälä P, Tommila M, Nurmi J. Intubation first-pass success in a high performing pre-hospital critical care system is not associated with 30-day mortality: a registry study of 4496 intubation attempts. Scand J Trauma Resusc Emerg Med. 2022;30. 10.1186/s13049-022-01049-7 PMC9677625 36411447

[31] Jarzebowski M, Estime S, Russotto V, Karamchandani K. Challenges and outcomes in airway management outside the operating room. Curr Opin Anaesthesiol. 2022;35:109–14. 35102045 10.1097/ACO.0000000000001100

[32] Sakles JC, Chiu S, Mosier J, Walker C, Stolz U. The Importance of First Pass Success When Performing Orotracheal Intubation in the Emergency Department. Acad Emerg Med. 2013;20:71–8. 23574475 10.1111/acem.12055 PMC4530518

[33] Davis DP, Lemieux J, Serra J, Koenig W, Aguilar SA. Preoxygenation Reduces Desaturation Events and Improves Intubation Success. Air Med J. 2015;34:82–5. 25733113 10.1016/j.amj.2014.12.007

[34] Papazian L, Klompas M, Luyt C-E. Ventilator-associated pneumonia in adults: a narrative review. Intensive Care Med. 2020;46:888–906. 32157357 10.1007/s00134-020-05980-0 PMC7095206

