# Supplementary material for: Non-invasive ventilation for preoxygenation during prehospital anaesthesia – a prospective observational study
Source: Scand J Trauma Resusc Emerg Med. 2025 Apr 23;33:67. doi: 10.1186/s13049-025-01386-3 (PMC12020282; doi:10.1186/s13049-025-01386-3)
Supplement: Supplementary file 1 — Additional file 1. [file 13049_2025_1386_MOESM1_ESM.docx]

# Survey of the use of non-invasive ventilation during anaesthesia

**Demographics**

Years of experience in anaesthesiology _______________________________

Years of experience in prehospital critical care _______________________________

Number of performed anaesthesia _______________________________

Number of performed prehospital anaesthesia _______________________________

Number of performed NIV preoxygenations _______________________________

**When to use NIV during preoxygenation**

Have you used non-invasive ventilation (Hamilton-T1) during Yes ⃞

preoxygenation? No ⃞

If “No”, you may skip the rest of the survey, but please answer why not?
_________________________________________________________________________________________________________________________________________________________________________________________________________________________________

When would you choose NIV over other methods for preoxygenation?

_________________________________________________________________________________________________________________________________________________________________________________________________________________________________

When do you deem NIV to be contra-indicated as a preoxygenation method:

_________________________________________________________________________________________________________________________________________________________________________________________________________________________________

**How to use NIV during preoxygenation**

Do you perform ventilations during apnoea with NIV? Always ⃞

When? Never ⃞

_________________________________________________________ Sometimes ⃞

_________________________________________________________

Do you apply adjunct nasal cannulas when using NIV? Always ⃞

When? Never ⃞

_________________________________________________________ Sometimes ⃞

_________________________________________________________

Do you use an oropharyngeal airway when using NIV? Always ⃞

When? Never ⃞

_________________________________________________________ Sometimes ⃞

_________________________________________________________

Do you use the standard settings suggested by the Hamilton-T1 for preoxygenation?

Yes, preoxygenation ⃞

Yes, preoxygenation with ventilations ⃞

No, I choose my own settings ⃞

If no, what would your choice of mode, rates and levels be? (Patient a male, 70kg, 180cm):

_________________________________________________________________________________________________________________________________________________________________________________________________________________________________

Do you have a minimum time you preoxygenate or other thresholds when you begin intubation?
_________________________________________________________________________________________________________________________________________________________________________________________________________________________________

How do you troubleshoot a potential difficult or failed NIV preoxygenation? What settings do you change? What adjuncts do you use? Do you change preoxygenation method or just deem preoxygenation failed and proceed to intubate?
_________________________________________________________________________________________________________________________________________________________________________________________________________________________________

**Thank you for your contribution!**

Harry Ljungqvist 040-7239349 [harry.ljungqvist@helsinki.fi](mailto:harry.ljungqvist@helsinki.fi)

Jouni Nurmi [jouni.nurmi@hus.fi](mailto:jouni.nurmi@hus.fi)
